# Rational Design of Peptide-Functionalized Surface Plasmon Resonance Sensor for Specific Detection of TNT Explosive

**DOI:** 10.3390/s17102249

**Published:** 2017-09-30

**Authors:** Jin Wang, Masaki Muto, Rui Yatabe, Takeshi Onodera, Masayoshi Tanaka, Mina Okochi, Kiyoshi Toko

**Affiliations:** 1 Research and Development Center for Taste and Odor Sensing, Kyushu University, 744 Motooka, Nishiku, Fukuoka 819-0395, Japan; yatabe@nbelab.ed.kyushu-u.ac.jp (R.Y.); toko@ed.kyushu-u.ac.jp (K.T.); 2 Department of Chemical Science and Engineering, Tokyo Institute of Technology, 2-12-1, O-okayama, Meguro-ku, Tokyo 152-8552, Japan; muto.masaki62@chugai-pharm.co.jp (M.M.); m_tanaka@chemeng.titech.ac.jp (M.T.); okochi@chemeng.titech.ac.jp (M.O.); 3 Department of Information Science and Electrical Engineering, Kyushu University, 744 Motooka, Nishiku, Fukuoka 819-0395, Japan; onodera@ed.kyushu-u.ac.jp

**Keywords:** rationally designed, TNT binding peptides, amino acid sequence, APTES, surface plasmon resonance, N-γ-maleimidobutyryl-oxysuccinimide ester

## Abstract

In this study, a rationally-designed 2,4,6-trinitrotoluene (TNT) binding peptide derived from an amino acid sequence of the complementarity-determining region (CDR) of an anti-TNT monoclonal antibody was used for TNT detection based on a maleimide-functionalized surface plasmon resonance (SPR) sensor. By antigen-docking simulation and screening, the TNT binding candidate peptides were obtained as TNTHCDR1 derived from the heavy chain of CDR1, TNTHCDR2 derived from CDR2, and TNTHCDR3 from CDR3 of an anti-TNT antibody. The binding events between candidate peptides and TNT were evaluated using the SPR sensor by direct determination based on the 3-aminopropyltriethoxysilane (APTES) surface. The TNT binding peptide was directly immobilized on the maleimide-functionalized sensor chip surface from N-γ-maleimidobutyryl-oxysuccinimide ester (GMBS). The results demonstrated that peptide TNTHCDR3 was identified and selected as a TNT binding peptide among the other two candidate peptides. Five kinds of TNT analogues were also investigated to testify the selectivity of TNT binding peptide TNTHCDR3. Furthermore, the results indicated that the APTES-GMBS-based SPR sensor chip procedure featured a great potential application for the direct detection of TNT.

## 1. Introduction

Due to the threat of global terrorism acts and the needs of homeland security, there has been a great increase in the development of sensitive, non-false alarm, portable, and cheap platforms to monitor ultra-trace levels of explosives. Those explosives—including 2,4,6-trinitrotoluene (TNT), 2,4-dinitrotoluene (DNT), 1,3,5-Trinitrobenzene (TNB), picric acid (PA), and hexogen (RDX)—result in severe effects on the environment and human health [1]. In the past decade, significant progress has been made by sensor researchers and engineers to develop more robust chemical sensors or biosensors for detecting these nitroaromatic compound explosives not only in aqueous solution, but also the vapor phase. Various materials and methods, such as single-walled nanotubes (SWNTs), nanowire coupled with a field effect transistor (FET), or nanoparticles with surface-enhanced Raman spectroscopy (SERS) have also been implemented for high-performance detection [2,3,4]. Meanwhile, the critical recognition elements including fluorescence quenching of conjugated polymer, cysteine-modified Au nanoparticles, APTES-based nanowire, antibodies, or peptides have been successfully employed for sensitive and selective explosive detection [5,6,7,8,9]. Among these, biomolecule recognition elements—peptides—are gaining tremendous attention for specific explosive detection because of their high affinity, long-term stability and sensitivity, and their capability of self-assembly on various materials (including nanoparticles and nanotubes) compared with other biological components, such as cells, proteins, and antibodies. Furthermore, they can be chemically engineered to obtain and design target peptides for specific demands [3,5,10,11]. All these advantages offer an ideal sensing element for explosive detection.

Based on our previous work, novel rationally-designed TNT binding peptides derived from an anti-TNT antibody were synthesized and screened by peptide array fluorescence measurement. The TNT candidate binding peptides were predicted and obtained from the complementarity-determining region (CDR variable region) in an anti-TNT antibody through antigen-dock simulation for selective detection [12]. In this study, we address the screening process of three rationally-designed TNT binding candidate peptides by immobilizing them on a maleimide-based surface plasmon resonance (SPR) sensor. This approach enables us to identify the TNT binding peptide that exhibits the best sensor performance (sensitivity, selectivity, reproducibility, linear range, etc.).

## 2. Materials and Methods

### 2.1. Materials and Reagents

TNT solution dissolved in pure DMF (*N*,*N*-dimethylformamide) was obtained from Chugoku Kayaku, Co., Ltd., Kure, Japan. The concentration of the received TNT solution was 10.3 mg/mL and was freshly diluted as required with phosphate-buffered saline (PBS, 0.1 M, pH 7.4) containing 0.05% Tween 20 (T) and 5% DMF before each measurement. Five kinds of TNT analogues, research and development explosive (RDX) solution dissolved in pure DMF (10.3 mg/mL) were purchased from Chugoku Kayaku, Co., Ltd., Kure, Japan. 2,4-Dinitrophenyl glycine (DNP-glycine), 2,4-DNT, 2,6-DNT, and 4-nitrobenzoyl-glycyl-glycine were purchased from Tokyo Chemical Industry, Tokyo, Japan. 3-Aminopropyltriethoxysilane (APTES) 99% was purchased from Sigma-Aldrich, St. Louis, MO, USA. N-γ-maleimidobutyryl-oxysuccinimide ester (GMBS) was purchased from Thermo Fisher Scientific, Rockford, IL, USA. L-cysteine was purchased from Sigma-Aldrich. The SPR Au chip was purchased from Biacore, GE, Uppsala, Sweden. Sodium hydroxide solution (NaOH) and potassium hydroxide solution (KOH) were purchased from Wako Pure Chemical Industries, Tokyo, Japan. All other chemicals were purchased either from Tokyo Chemical Industry, Tokyo, Japan or Wako Pure Chemical Industries, Tokyo, Japan. All aqueous solutions were prepared by MilliQ deionized water (18 MΩ) from MilliQ-system (Millipore Corporation, Billerica, MA, USA).

### 2.2. Rational Design of TNT Binding Peptide from Anti-TNT Antibody

According to our previous work, three rationally-designed peptide sequences in the heavy chain of the complementarity determining region (CDR) in anti-TNT antibody were obtained [12]. TNT binding peptide was screened from anti-TNT antibody produced by hybridoma cell. The amino sequence was identified to be GYSITSHY (HCDR1), ISYSGST (HCDR2), ARGYSSFIYWFFDF (HCDR3). The three candidate TNT binding peptides were used for surface plasmon resonance through direct measurement. Cysteine was added at the carboxyl-terminal of each candidate peptide for the thiol-maleimide interaction.

### 2.3. Instrumentation

The direct evaluation of TNT binding peptides was carried out by a Biacore X100 instrument (GE Healthcare, Amersham, Great Britain). The Biacore X100 instrument is an automatic system which is designed for kinetic/affinity screening and characterization, immunogenicity, and low molecular weight (LMW) interaction analysis with low baseline noise (<0.1 resonance units (RU), low baseline drift (<0.3 RU/min), high precision, only 32–120 μL sample volume consumption, and data collection rate at 1 Hz. The analysis temperature could be maintained at 25 ± 1 °C. An increase in resonance units (RU) corresponds to an increase in the amount of bound target on the surface. One resonance unit is determined as 0.0001° of the resonance angle shift, and is equivalent to a change in mass of 1 pg·mm^−2^ on the surface. NaOH solution was used for the regeneration of the sensor surface. In most of the experiments, surface-bound protein or antibodies were completely dissociated when 50 mM NaOH was flowed over the surface.

### 2.4. Fabrication Procedure of Sensor Chip Surface

The SIA Kit Au (Biacore, GE, Uppsala, Sweden) contains sensor chips covered with a 50-nm-thick unmodified gold layer, and was used for the fabrication of multistep immobilization of various films on surface. The sensor chip was cleaned in a mixed solution of Milli-Q water, ammonia solution, and hydrogen peroxide with a 10:2:2 volume ratio at 90 °C for 20 min. After the cleaning process, the Au chip was cleaned with 90 μL KOH (1% *w*/*v*) for 5 min to generate hydroxyl groups followed by extensive washing with MilliQ deionized water. The chip was then incubated with 100 μL of 2% (*v*/*v*) APTES for 1 h at room temperature (RT) in a fume hood followed by five washes with MilliQ deionized water. The procedure functionalized the Au surface with APTES and generated amine groups on its surface [13,14,15]. Then, the APTES-based Au chip was incubated with 8 mM GMBS for 1 h to form a maleimide moiety. Thiol-modified peptides can easily attach onto a maleimide functionalized surface. After that, 2000 ppm of each peptide, prepared with 90% DMF solution in PBST (PBS containing 0.05% Tween 20) buffer (pH 7.4) was immobilized on the NHS ester surface for 2 h and the surface was blocked with 50 mM L-cysteine for 30 min. After rinsing with Milli-Q water, the chips were blown to dry under nitrogen. The fabricated chip was stored in a sealed bag filled with nitrogen for use at 4 °C. A concentration series of TNT solutions (4.0 ppm, 7.9 ppm, 15.7 ppm, 31.4 ppm, 62.7 ppm, 125.4 ppm, 250.8 ppm, and 501.5 ppm) was injected in triplicate for 180 s at a 10 μL/min rate, respectively. The binding responses were concentration-dependent and the sensor surface was regenerated by using 5 mM NaOH for 12 s since the interactions are rapid and mild regeneration is required. For preparation of TNT solution and running buffer of the Biacore system, 5% DMF was added to maintain the analyte solubility and eliminate the bulk effects. Before each measurement, the system was primed at least three times using a running buffer and three injections with buffer solution at the beginning of each binding cycle before analyte sample injection. After each measurement, the Biacore system was cleaned by using 50% DMSO and 10% DMSO to wash out the sticky TNT molecule residue to avoid interference.

## 3. Results and Discussion

### Screening of TNT-Binding Peptides among HCDR Using Maleimide-Based Sensor

**Figure 1:**
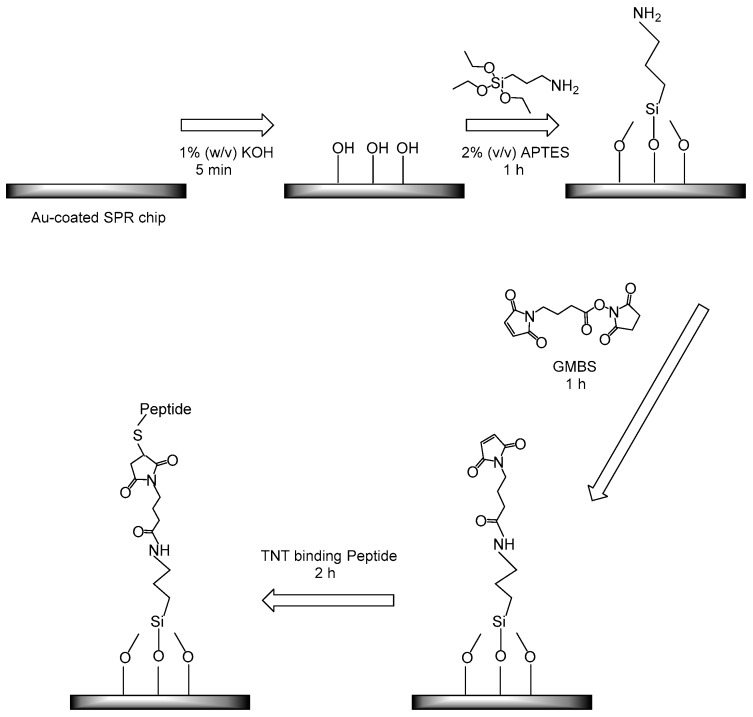
Immobilization procedure of the 2,4,6-trinitrotoluene (TNT) binding peptide on the surface of the surface plasmon resonance (SPR) Au-chip. APTES: 3-aminopropyltriethoxysilane; GMBS: N-γ-maleimidobutyryl-oxysuccinimide ester.

The obtained TNT candidate peptides were covalently bound to the maleimide-based SPR sensor chip via thiol-modified peptides (Figure 1). For LMW compound detection using an SPR sensor, some detection methods—including the displacement method and competitive inhibition method—have been reported and developed by using the antibody–antigen reaction in our previous work [16,17,18,19]. For the development of a novel detection system for TNT, it was a challenge that rationally-designed peptides were directly immobilized on the sensor chip surface for TNT detection, which required a high binding capacity to LMW compounds. Compared with our preliminary results of using the CM5 chip and self-assembled monolayer-based peptide-functionalized SPR gold chip (data not shown), the maleimide-based sensor surface chip is promising for screening TNT binding candidate peptides since APTES are short organic molecules approximately 0.6 nm in length—much shorter than the CM5 dextran matrix (100 nm). Such shorter attachment intermediates could further improve the signal amplification and sensitivity enhancement of SPR assays. Three candidate peptides HCDR1, HCDR2, and HCDR3 were diluted with 90% DMF solution in PBST buffer (pH 7.4) for immobilization due to the high percentage of hydrophobic amino acids in the sequence.

**Figure 2:**
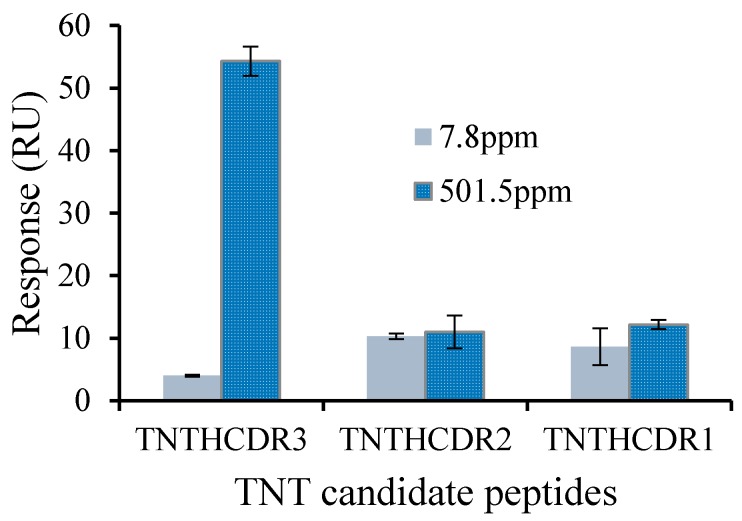
Screening TNT binding peptide among candidate peptides TNTHCDR1, TNTHCDR, and TNTHCDR3 using an APTES-based chip. The error bar indicates the calculated standard deviation (*n* = 3). RU: resonance units.

**Figure 3:**
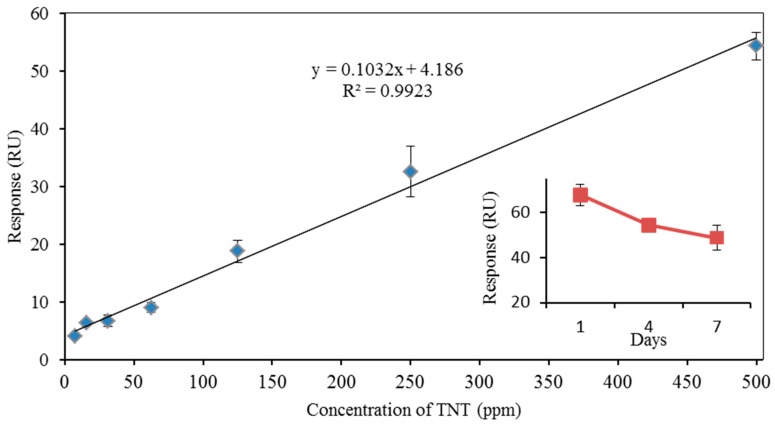
A plot of the response of the APTES-GMBS-based sensor chip immobilized with the TNT binding peptide TNTHCDR3 corresponding to various TNT concentrations (inset: reproducibility of the sensor chip). The error bar indicates the calculated standard deviation (*n* = 3).

Among three HCDR sequences, only HCDR3 contained the amino acid tryptophan (W), which plays a very important role binding to TNT by π-electron-mediated effects from the planar structure of the aromatic amino acids [11,20], predicted to be a TNT-binding peptide by TNT docking simulation and peptide array measurement. The results in Figure 2 showed that the candidate TNTHCDR3 exhibited the highest binding signal to TNT compared with the other two candidate peptides TNTHCDR1 and TNTHCDR2, indicating that TNTHCDR3 was successfully synthesized and derived from an anti-TNT antibody for direct TNT detection, which agreed with the results of the predication and peptide array measurement [12]. A plot of the response of the APTES-GMBS-based sensor chip immobilized with TNT binding peptide TNTHCDR3 corresponding to various TNT concentrations is obtained in Figure 3. The linear range is from 7.8 ppm to 501.5 ppm (R^2^ = 0.9923) with the limit of detection (LOD) calculated at 1.35 ppm (3σ). The results were comparable with the reported peptide-based quartz crystal microbalance (QCM) and square wave voltammetry sensor for direct TNT detection [5,21].

**Figure 4:**
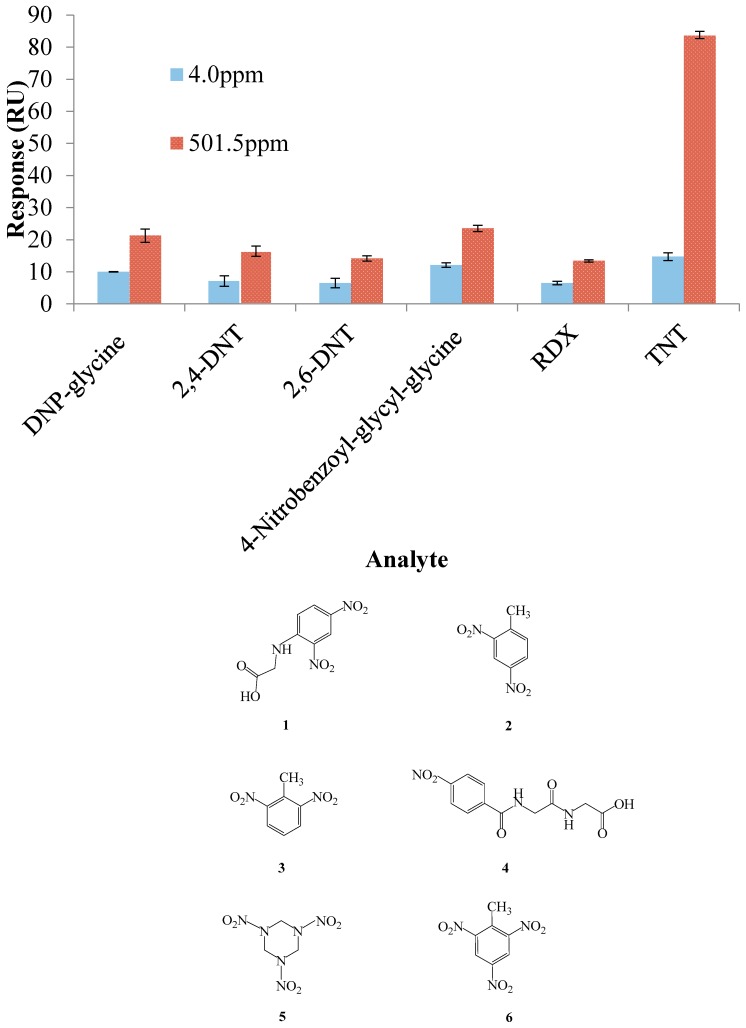
The response of TNTHCDR3 anchored SPR Au sensor chip towards 4.0 ppm (**blue**) and 501.5 ppm (**red**) solutions of 2,4-dinitrophenyl glycine (DNP-glycine) (**1**), 2,4-dinitrotoluene (2,4-DNT) (**2**), 2,6-DNT (**3**), 4-nitrobenzoyl-glycyl-glycine (**4**), research and development explosive (RDX) (**5**), and TNT (**6**). The error bar indicates the calculated standard deviation (*n* = 3).

In our previous work [16,17,18,19], the displacement method and competitive inhibition method based on an SPR immunosensor were exploited for the detection of TNT. Although these methods offered more sensitive detection (ppt–ppb) through an antibody-based SPR immunosensor, they suffered from complicated TNT antibody preparation, large amounts of consumption of extremely expensive reagents, and surface damage caused by regeneration with the strong regeneration solution. Unlike the TNT antibody, the TNT binding peptide could be easily chemically synthesized according to the obtained amino acid sequence with excellent storage stability. The inset of Figure 3 shows the reproducibility of the sensor chip. The TNT concentration was chosen at 501.5 ppm, which required regeneration. The binding response of the sensor was decreased mainly because of the degraded peptide activity caused by surface regeneration. The selectivity of the rationally-designed TNT binding peptide TNTHCDR3 was also investigated (Figure 4). The results clearly showed that the TNTHCDR3 peptide has a strong preference for binding TNT over five kinds of TNT analogues: DNP-glycine, 2,4-DNT, 2.6-DNT, RDX, and 4-nitrobenzoyl-glycyl-glycine. The highest concentration allowed of these analogues was 501.5 ppm and the lowest concentration was 4.0 ppm. Analysis of these results revealed low non-specific binding and high specific binding between TNT and TNTHCDR3, demonstrating that the TNT binding peptide was successfully rationally designed and screened through the other two TNT candidate peptides. Furthermore, to our knowledge, this is the first report that uses a TNT binding peptide-based SPR sensor for direct measurement of TNT. The results shown above illustrate that the TNTHCDR3 peptide-anchored SPR sensor was successfully fabricated for TNT explosive detection, which opens up development avenues for future LMW detection.

## 4. Conclusions

The present study has demonstrated that rationally-designed TNT binding peptides predicted and obtained from anti-TNT monoclonal antibody were screened and identified for TNT explosive detection using maleimide-based SPR sensor through direct measurement. TNTHCDR3 was determined as TNT binding peptide with high-selectivity over five kinds of TNT analogues. The SPR evaluation results exhibited ppm-level sensitivity for direct TNT determination since it is a challenge for direct LMW compound detection at low concentrations. We hope, in the near future, to create a more sensitive and better-selective platform for TNT detection by using this TNTHCDR3 binding peptide.
